# CDK4/6 Inhibitor-Mediated Cutaneous Toxicity in Breast Cancer

**DOI:** 10.7759/cureus.105839

**Published:** 2026-03-25

**Authors:** Juan M Alcantar, Fukai L Chuang, David D Kim

**Affiliations:** 1 Hematology and Medical Oncology, University of California Los Angeles David Geffen School of Medicine, Los Angeles, USA; 2 Medicine, University of California Los Angeles David Geffen School of Medicine, Los Angeles, USA

**Keywords:** breast cancer, bullous dermatitis, cdk4/6 inhibitors, rash, topical corticosteroids

## Abstract

Cyclin-dependent kinase 4 and 6 (CDK4/6) inhibitors are currently in widespread use for the treatment of both advanced metastatic and early-stage hormone receptor (HR)-positive, human epidermal growth factor receptor (HER2)- negative breast cancer. Clinical studies in the metastatic setting have demonstrated a survival benefit with the use of ribociclib. Both abemaciclib and ribociclib have shown significant improvements in invasive disease-free survival in high-risk early-stage breast cancer. Hematologic adverse events are well characterized with the three currently approved CDK4/6 inhibitors, namely palbociclib, abemaciclib, and ribociclib. Among non-hematologic toxicities, there is a paucity of information regarding cutaneous adverse events related to these three medications. In this case report, we seek to describe the incidence, time to onset, and management of CDK4/6-mediated cutaneous adverse events.

## Introduction

Breast cancer is the most frequent type of cancer in women [1]. In the year 2025, there were an estimated 316,950 new cases of invasive breast cancer diagnosed in women, with an estimated 42,170 breast cancer deaths [2]. The traditional clinical sub-classification of breast cancer is based on results of immunohistochemical testing for estrogen receptor (ER), progesterone receptor (PR), and human epidermal growth factor receptor (HER2). Both ERs and PRs are ligand-activated nuclear transcription factors [3]. Hormone receptor (HR)-positive breast cancer is defined by the expression of ER, PR, or both [4]. Guidelines provided by the American Society of Clinical Oncology (ASCO)/College of American Pathologists defined ER and/or PR positivity using a threshold of ≥ 1% immunohistochemically reactive tumor cell nuclei [5]. Based on Surveillance, Epidemiology, and End Results (SEER) data for 2018-2022, the age-adjusted rate for new cases of HR-positive breast cancer was 103.6 per 100,000 women, making this type of breast cancer the most common [6].

In women with HR-positive, HER2-negative breast cancer, endocrine therapy has been prominent in the frontline treatment of breast cancer. Agents such as the selective estrogen receptor modulator (SERM) tamoxifen, as well as aromatase inhibitors (AIs), have been instrumental in improving clinical outcomes for both advanced metastatic and early-stage disease. The addition of oral cyclin-dependent kinase 4 and 6 (CDK 4/6) inhibitors to endocrine therapy marked a pronounced paradigm shift in the management of breast cancer [7]. As is true with novel oral oncologic medications, new and previously unrecognized toxicity signals have emerged with the routine use of CDK 4/6 inhibitors, which warrant further study in order to provide optimal management guidelines. It is recognized that orally targeted cancer-treating medications can have a detrimental effect on quality of life [8].

In this case report, we describe one of the potential clinical presentations of CDK 4/6 inhibitor-associated cutaneous toxicity. We also provide a brief overview of the molecular and cellular pathways affected by CDK 4/6 inhibitors. We describe the prevalent use and efficacy of CDK 4/6 inhibitors in the adjuvant and metastatic setting for breast cancer. In this context, we describe the heterogeneity of cutaneous adverse events in these patient populations as well as therapeutic management options.

## Case presentation

A 68-year-old postmenopausal female was diagnosed with early-stage bilateral breast cancer after presenting with bilateral breast masses. On presentation, ultrasound-guided biopsies were performed of bilateral breast masses. Right breast pathology showed grade 1 invasive ductal carcinoma with lobular features, ER 90%, progesterone 80%, HER2 1+ by immunohistochemistry (IHC), and no HER2 amplification by fluorescence in situ hybridization (FISH). The left breast biopsy showed grade 1 invasive mammary carcinoma with lobular features. Biopsy of the left axillary lymph node was also consistent with grade 1 invasive ductal carcinoma with lobular features. The staging CT scan of the chest, abdomen, and pelvis was notable for bilateral breast masses and prominent left axillary lymph nodes.

The patient underwent a left modified radical mastectomy with a concurrent right simple mastectomy in conjunction with a right sentinel lymph node biopsy. Surgical pathology on the right breast showed a 3.2 centimeter (cm) grade 2 invasive carcinoma with two out of four lymph nodes positive for metastatic breast cancer, pathological stage T2N1aMx. Surgical pathology from the left breast showed a 6.8 cm grade 2 invasive carcinoma with nine of 17 lymph nodes positive for metastatic breast cancer, pathological stage T3N2aMx.

Past medical history was notable for well-controlled hypertension and hypothyroidism. The patient had had a hysterectomy secondary to uterine fibroids with ovaries in situ. Hereditary germline testing did not reveal any pathogenic mutations. The patient completed adjuvant chemotherapy with sequential dose-dense Adriamycin-cyclophosphamide (AC) followed by dose-dense paclitaxel. Subsequently, the patient also completed bilateral chest wall radiation therapy. The patient started a course of adjuvant endocrine therapy with letrozole. Given the high risk of breast cancer recurrence, the patient was also started on a planned three-year course of adjuvant ribociclib and zoledronic acid every six months.

**Figure 1:**
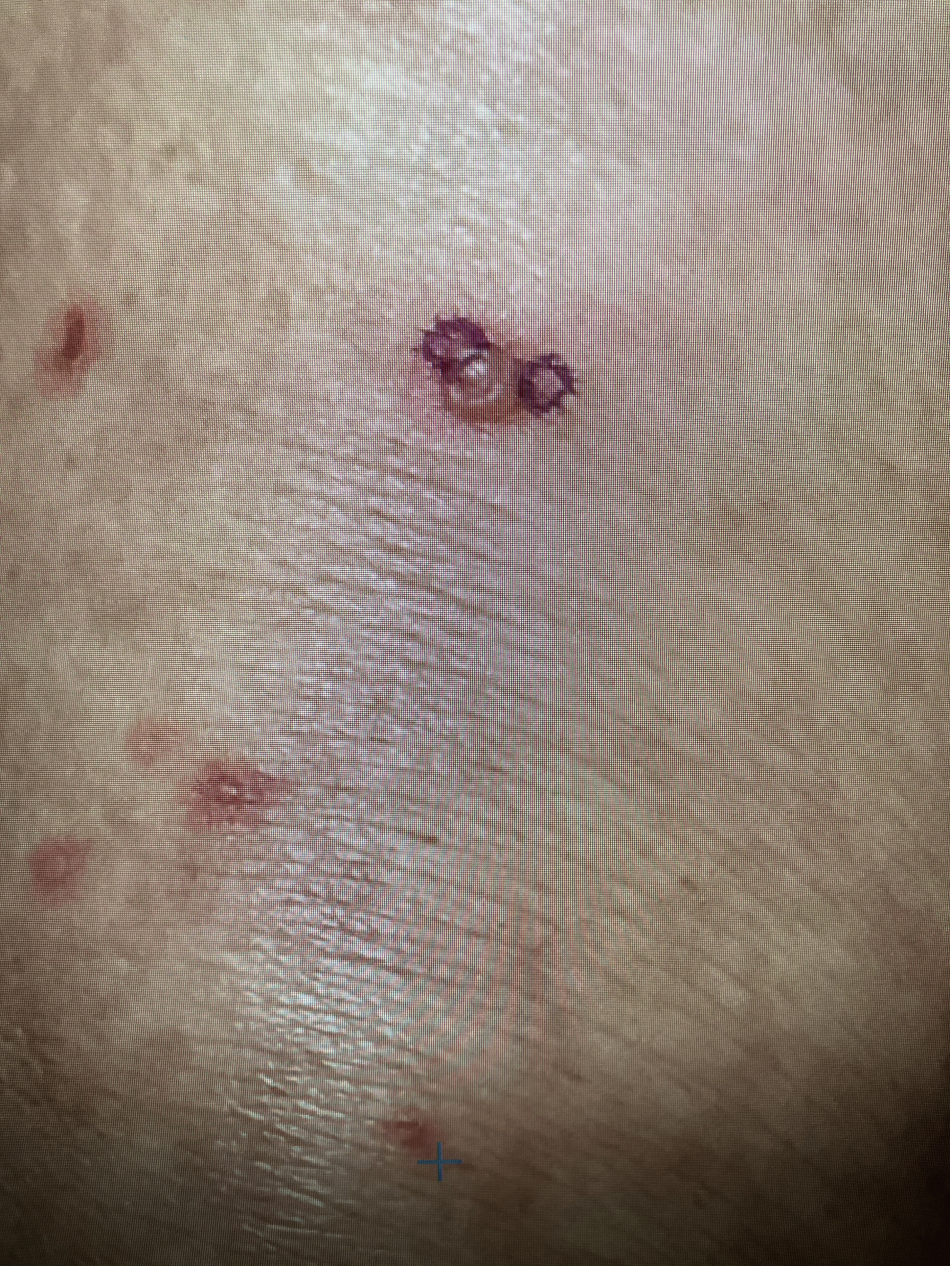
Right anterior thigh bullous rash.

**Figure 2:**
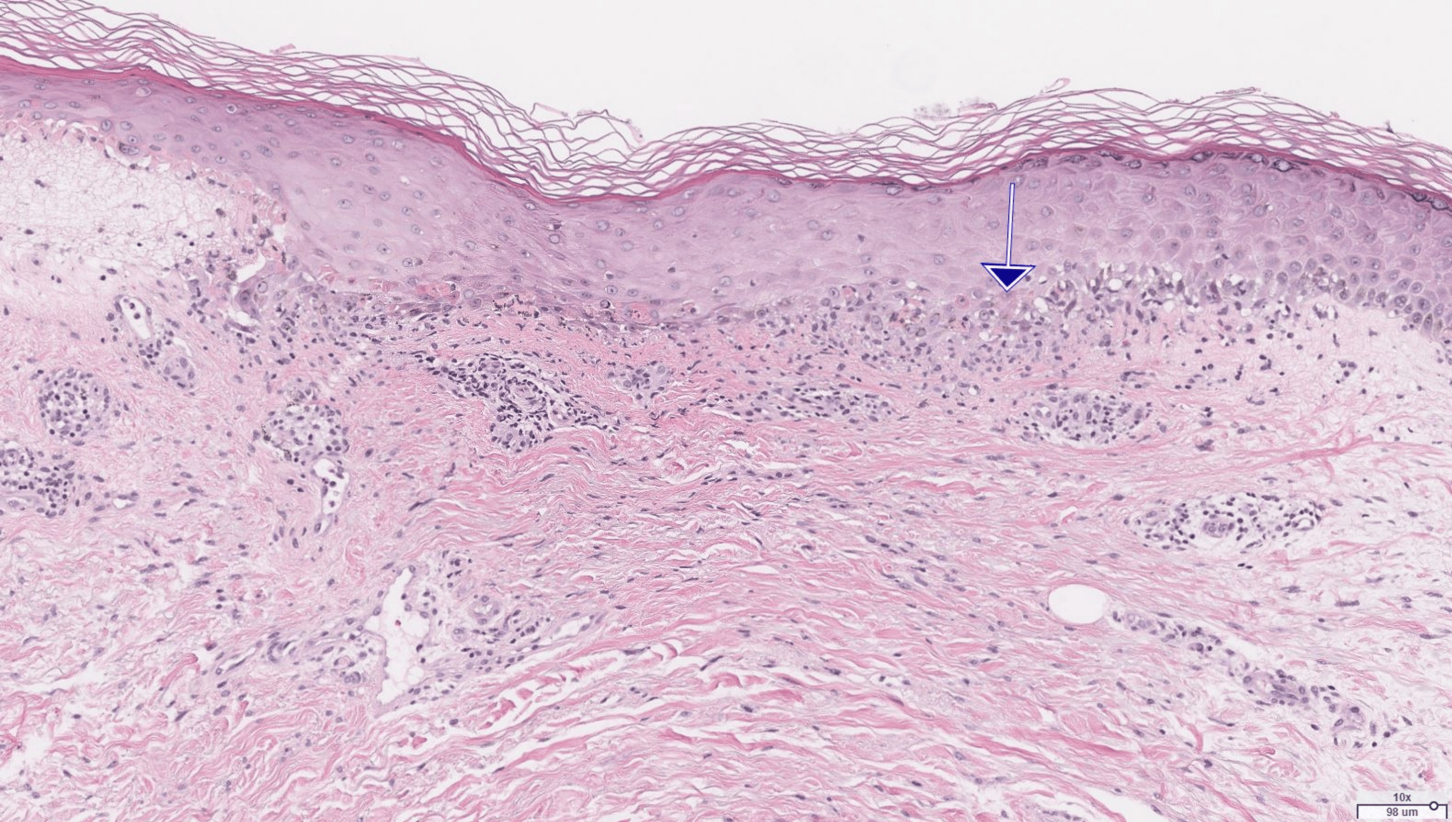
Arrow pointing to area of interface changes with necrotic keratinocytes (10x H&E).

The patient's clinical course was uncomplicated until about one year into ribociclib treatment. At this point, the patient developed a pruritic blistering rash involving the trunk as well as all four extremities (Figure 1). The patient was seen in consultation by dermatology and had punch biopsies of the bilateral anterior thigh rash. Pathology from both sites showed interface dermatitis with epidermal necrosis and subepidermal/intraepidermal clefting, superficial dermal perivascular and interstitial inflammation containing lymphocytes and neutrophils (Figure 2). Furthermore, the direct immunofluorescence study was negative, which excluded drug-induced bullous pemphigoid. Overall, clinicopathologic findings were consistent with bullous drug-associated eruption, favoring fixed drug or erythema multiforme-like drug eruption. The patient experienced complete resolution of symptoms and rash with topical clobetasol cream 0.05% twice daily. 

Upon resolution of the rash, letrozole was reintroduced with no rash recurrence. Given the high risk of breast cancer recurrence, the patient was started on adjuvant abemaciclib with a plan of two years of treatment. Unfortunately, the patient had a recurrence of the pruritic blistering rash, and abemaciclib was discontinued. The patient continues to be breast cancer-free without recurrence of cutaneous toxicity on single-agent letrozole.

## Discussion

CDK 4/6 inhibitors are currently essential oncologic drugs for the management of advanced metastatic HR-positive, HER2-negative breast cancer in combination with endocrine therapy. Palbociclib was approved by the Food and Drug Administration (FDA) in 2017 based on results of the PALOMA-2 (PAlbociclib: Ongoing Trials in the MOMAnagement of Breast Cancer-2) clinical trial [9], which showed an improvement in progression-free survival (PFS) in combination with letrozole in comparison to the placebo/letrozole arm. Similarly, both ribociclib and abemaciclib are also FDA-approved for use in combination with endocrine therapy in advanced HR-positive, HER2-negative breast cancer. In the MONARCH-3 (A Study of Nonsteroidal Aromatase Inhibitors Plus Abemaciclib in Postmenopausal Women With Breast Cancer) clinical trial [10], abemaciclib in combination with a non-steroidal AI showed a clinically meaningful improvement in overall survival (OS). In both MONALEESA-2 (Mammary ONcology Assessment of LEE011’s (Ribociclib’s) Efficacy and SAfety-2) [11] and MONALEESA-7 [12], ribociclib showed a significantly longer OS when given in combination with a non-steroidal AI. 

Conversely, in the adjuvant setting, both ribociclib and abemaciclib are FDA-approved for use in HR-positive, HER2-negative breast cancer deemed at high risk for recurrence. In the monarchE trial, the addition of two years of adjuvant abemaciclib to endocrine therapy in high-risk early breast cancer resulted in improvement in both invasive disease-free survival (iDFS) and distant recurrence-free survival (DRFS). At five years, there was an absolute improvement in iDFS of 7.6% and 6.7% in DRFS, respectively [13]. In the monarchE trial, high risk was defined as having ≥4 positive lymph nodes, or one to three positive lymph nodes and at least one of the following: tumor size ≥5 cm, histologic grade 3, or central Ki-67 ≥20 percent. In the NATALEE (New Adjuvant Trial with Ribociclib (LEE011)) trial, patients with stage IIA and III breast cancer were randomly assigned to a non-steroidal AI with or without adjuvant ribociclib for a total duration of three years. This trial also demonstrated an improvement in the primary endpoint of iDFS with an absolute improvement of 3.6% with combination treatment, non-steroidal AI plus ribociclib vs. non-steroidal treatment alone [14].

The cell cycle is the fundamental process by which cell division occurs in eukaryotic cells. The cell cycle directs an individual cell through a sequence of events that ultimately come to an end in mitosis and the production of two daughter cells. There are four stages of the cell cycle known as G1, S, G2, and M phases [15]. G1 and G2 are gaps that occur prior to DNA synthesis (S phase) and mitosis (M phase), respectively. The progression of an individual cell through the cell cycle is dependent on CDKs and the cyclin proteins. CDKs are especially important for a cell’s progression through the cell cycle because their inactivation prevents mitosis. In particular, CDK4 and CDK6 are essential in mediating progression through the G1 gap, at which time the cell prepares to initiate DNA synthesis [16]. Activation of CDK4 and CDK6 is dependent on D-type cyclins D1, D2, and D3. Activated CDK4 and CDK6 result in the phosphorylation and inhibition of the retinoblastoma tumor suppressor Rb. This leads to Rb dissociation from E2F transcription factors, thereby driving expression of E2F-target genes that initiate DNA replication [17]. 

A significant therapeutic role for CDK 4/6 inhibitors is now well established in routine clinical oncology practice. As is true with other oral therapeutic agents, CDK 4/6 inhibitors are associated with particular toxicity profiles. In regard to common adverse events, palbociclib is associated with all-grade adverse event rates of 76% and 95% for thrombocytopenia and neutropenia, respectively [18]. Abemaciclib is associated with a 20% rate of grades 3 and 4 diarrhea and a 90% rate of all grades of diarrhea. Cytopenias are also common with ribociclib, which itself is associated with an all-grades 8% rate of corrected QT interval prolongation. 

Cutaneous adverse events are not commonly noted in the day-to-day use of the three currently approved CDK 4/6 inhibitors. In a retrospective, multi-center study, Sollena et al. were able to provide further insight into potential cutaneous adverse events that may be encountered in clinical practice [19]. The study included 79 patients with metastatic breast cancer, HR-positive, and HER2-negative, receiving treatment with an oral CDK 4/6 inhibitor. In total, 165 cutaneous adverse events were noted, with pruritus being most common at 62%, followed by alopecia at 32%, and eczematous rash at 31%. Median time to onset was six months for palbociclib, 6.5 months for ribociclib, and nine months for abemaciclib. Other less common cutaneous adverse events can be seen with this class of medications. Vitiligo-like lesions (VLLs) were noted in 5% of patients receiving palbociclib and in 20% of patients with ribociclib. CDK 4/6 inhibitors can also be associated with more severe and potentially life-threatening cutaneous toxicity. Although not frequent, cases of toxic epidermal necrolysis, Stevens-Johnson syndrome, bullous dermatitis, and drug-induced cutaneous lupus erythematosus have been described. 

CDK 4/6 inhibitors are now also a mainstay treatment for both high-risk early-stage as well as advanced metastatic breast cancer that is HR-positive, HER2-negative. Multiple prospective studies have now provided insight into the expected adverse events profile. Dermatologic toxicity accounts for up to 15% of all reported adverse events, which are mild to moderate with 1% grade 3 or more [20]. Management of CDK 4/6 inhibitors' cutaneous toxicity involves a multidisciplinary approach involving medical oncology as well as dermatology specialists. Temporary treatment interruption may be indicated in some cases, although reassuring is the fact that the rate of permanent discontinuation is low at < 5% of cases [20]. In general, skin-directed therapy with topical high-potency corticosteroids represents the cornerstone of management of CDK 4/6 inhibitors' cutaneous toxicity. In the retrospective, multicenter cohort study by Sollena et al., a complete or partial response was noted in 65 out of 79 cases. In this particular study, a small number of patients with moderate to severe cutaneous reactions received systemic therapy with oral prednisone or Ultraviolet A/B phototherapy. 

## Conclusions

As noted, a more comprehensive understanding of the biologic role of CDKs within the cell cycle has led to the development of a new class of oncologic drugs in the form of CDK 4/6 inhibitors. This novel type of oral medication has now made significant improvements in clinically meaningful endpoints in both early-stage and advanced metastatic breast cancer that is HR-positive and HER2-negative. Medical oncologists are typically well versed in the incidence and management of both hematologic and gastrointestinal toxicity with this class of drugs. Cutaneous adverse events are also common and manageable, leading to improved clinical outcomes in this patient population.
